# Atrial Fibrillation Underlies Cardiomyocyte Senescence and Contributes to Deleterious Atrial Remodeling during Disease Progression

**DOI:** 10.14336/AD.2021.0619

**Published:** 2022-02-01

**Authors:** Ailiya Adili, Xiyu Zhu, Hailong Cao, Xinlong Tang, Yali Wang, Junxia Wang, Jian Shi, Qing Zhou, Dongjin Wang

**Affiliations:** Department of Cardio-Thoracic Surgery, Nanjing Drum Tower Hospital, the Affiliated Hospital of Nanjing University Medical School, Nanjing, Jiangsu, China

**Keywords:** Atrial Cardiomyocyte Senescence, Atrial Fibrillation, Atrial Remolding, DNA Damage

## Abstract

Accelerated senescence is triggered by key mediators of arrhythmogenic substrates and contributes to atrial fibrillation (AF). We sought to understand senescence in AF and the extent to which it aggravates the AF process. Twenty-six AF patients undergoing open-heart surgery were included, and 12 patients with sinus rhythm served as controls. Another cohort included 120 consecutive persistent AF patients with valvular heart diseases. HL-1 atrial myocytes were tachypaced (TP) to simulate experimental AF. Compared with sinus rhythm, left atrial appendages (LAAs) with AF presented a significantly increased positive area of cellular senescence, with upregulated expression of p16, p21 and p53. Next, p21 mRNA was increased in patients with AF recurrence compared with that in patients without recurrence. In multivariate analysis, p21 (OR: 2.97; 95% CI: 1.65-5.34; P<0.001) was a significant independent predictor of AF early recurrence. Interestingly, TP induced HL-1 atrial myocyte senescence in vitro, accompanied by a marked increase in the senescence-associated secretory phenotype (SASP) and altered the expression of sarcoplasmic reticulum (SR)-related proteins. Suppression of p21 by siRNA reduced TP induced cell senescence and IL-1β, IL-6 elevation, and partly changed SR-related proteins expression. Moreover, we show that the level of γH2AX, a marker of DNA damage, was higher in AF patients than in sinus rhythm controls. Similarly, an increase in γH2AX levels was observed following TP. AF underlies cardiomyocyte senescence and contributes to deleterious atrial remodeling during disease progression. This finding may help facilitate the search for new therapeutic approaches for antiaging therapy for AF.

Atrial fibrillation (AF) is the most frequent arrhythmia in clinical practice and is closely associated with increased cardiovascular morbidity and mortality [1]. The accumulation of damaged cells and decreased cellular function in the atria due to various cardiac diseases and conditions cause atrial remodeling, contributing to the initiation and perpetuation of AF [2]. AF, in turn, induces cell damage and atrial remodeling, increasing the susceptibility of the atria to AF induction and maintenance (i.e., “AF begets AF”)[3].

Accumulating evidence has shown that the incidence and prevalence of AF increase with age [4, 5]. Moreover, aging is an important risk factor for AF recurrence [6]. Age-dependent changes in the atrium contribute to atrial dysfunction and increase the risk of AF development in the elderly [7, 8]. As the population continues to age, much attention has been dedicated to understanding the essential roles and possible mechanisms of aging in human disease pathophysiology [8]. Indeed, the close link between AF and aging is currently under intense investigation. However, the cellular and molecular mechanisms involved still need to be fully established.

At the cellular level, aging is characterized by cellular senescence, a state of irreversible cell cycle arrest and loss of specialized cellular functions [9]. Senescent cells accumulate with age; as a result of genotoxic stress or various chronic diseases, they contribute to tissue aging and have been implicated in age-related tissue dysfunction because of the accumulation of damaged cells at sites of tissue injury and remodeling [9-11]. Premature cellular senescence is characterized by increased expression of cell cycle inhibitors, including p16, p21, and p53, and increased senescence-associated β-galactosidase (SA-β-gal) activity [12-14]. Additionally, a senescence-associated secretory phenotype (SASP), in which a significant amount of proinflammatory cytokines and signaling molecules is released, leads to changes in cellular structures and functions [9]. SASP has also been implicated in cardiovascular aging and disease progression by influencing neighboring cells or the microenvironment [15]. Therefore, activation of the p53 and/or p16 pathways, increased SA-β-gal activity, higher levels of DNA damage and the acquisition of SASP are widely employed to confirm cellular senescence [14].

Premature cell senescence contributes to age-related myocardial dysfunction in postmitotic tissues [16]. Emerging evidence suggests that a short telomere length, a hallmark of biological aging, is associated with AF incidence [17], indicating that premature aging is a common pathogenic mechanism of AF. Recently, researchers have investigated the link between AF and cell senescence, showing that endothelial or fibroblast senescence may pave the way for adverse atrial remodeling in AF by promoting proinflammatory, prothrombotic, and profibrotic responses [18-20]. Premature aging and senescence are associated with atrial remodeling in AF, highlighting the close relationship between AF and cell senescence. However, this finding is insufficient to explain how atrial cell senescence may contribute to AF pathophysiology. In AF, the mechanisms that drive cellular senescence and the role of senescence in atrial remodeling remain to be elucidated. In this study, we sought to understand senescence in AF and the extent to which it aggravates the AF process.

## MATERIALS AND METHODS

### HL-1 cardiomyocyte culture, rapid electrical stimulation, transfections and treatments

HL-1 cardiomyocytes were obtained from Dr. William Claycomb (Louisiana State University, New Orleans) and cultured in complete Claycomb medium (Sigma; 51800C) supplemented with 10% fetal bovine serum (Wisent), 100 U per ml penicillin (Wisent), 100 µg per ml streptomycin (Wisent), 4 mM L-glutamine (Sigma), 0.3 mM L-ascorbic acid (Sigma), and 100 µM norepinephrine (Sigma). HL-1 cardiomyocytes were cultured on cell culture plastics or glass coverslips coated with 0.02% gelatin (Sigma) in a humidified atmosphere of 5% CO_2_ at 37°C [21].

Gelatin/fibronectin-coated six-well culture dishes were seeded with HL-1 cells (≥3×10^5^ cells). When the cells reached confluence, they were subjected to rapid electrical stimulation to achieve a 5-fold rate increase, as observed in clinical AF (5 HZ; 3 V; pulse duration of 5 ms) using a C-Pace100 culture pacer (IonOptix) for 12 or 24 h[22, 23]. Tyrode solution medium (137 mmol/l NaCl, 5.4 mmol/l KCl, 1.2 mmol/l MgSO_4_, 1.8 mmol/l CaCl_2_, 1.2 mmol/l KH_2_PO_4_, 10 mmol/l glucose and 10 mmol/l HEPES, pH adjusted to 7.4 with NaOH) was used during electrical stimulation. Cells cultured in normal Tyrode solution without electrical stimulation for 12 or 24 h were used as the normal control group, and electrical stimulation (1 HZ; 3 V; pulse duration of 5 ms) using a C-Pace100 culture pacer (IonOptix) at the indicated times was used as the pacing control group [22,24].

SiRNAs (Small interfering RNA) for p21 and their control siRNAs were purchased from RiboBio and transfected into HL-1 cells using Lipofectamine 3000 (Thermo Fisher) following the manufacturer’s protocol. For the AngII experiment, after the cultures reached confluence in growth medium (Claycomb medium), the cells were transferred to serum-free Claycomb medium and incubated with 1µm/l AngII (Sigma) for the indicated times [24].

### Patients

Between September 2018 and June 2019, all patients undergoing open-heart surgery at our hospital (Nanjing Drum Tower Hospital, China) were screened for participation in this study. Twelve patients with paroxysmal short-term AF (SAF) and 14 with long-standing permanent AF (LAF) undergoing open-heart surgery were included; 12 patients with sinus rhythm served as controls. Another cohort included 120 consecutive persistent AF patients who required the RF maze procedure concomitant with mitral valve surgery. A preoperative 7-day Holter assessment and comprehensive transthoracic echocardiographic examination were performed to establish that all the patients had persistent valvular AF. Persistent AF was defined as any AF episode lasting longer than 7 days or requiring termination by cardioversion. Patients with relevant comorbidities (malignancies and chronic inflammatory diseases) were excluded.

Before surgery, the patient characteristics were collected. All the patients’ baseline characteristics, including medical history, transthoracic echo-cardiography, 12-lead electrocardiography (ECG) recording, and routine hematological and biochemical blood tests before surgery, were collected following admission. Left atrial appendage (LAA) tissue from AF and sinus rhythm patients undergoing open-heart surgery was analyzed for the characteristics of premature cell senescence (patients aged >60 years were excluded to avoid the confounding effects of age on disease). Next, 120 consecutive persistent AF patients with valvular heart diseases were followed up for 3 months after the maze procedure.

### Human sample preparation

Atrial appendages were immediately snap-frozen in liquid nitrogen and stored at -80°C. All patients provided written informed consent and were admitted to the Affiliated Drum Tower Hospital of Nanjing University Medical School. The study complied with the principles outlined in the Declaration of Helsinki and was approved by the institutional review board of Nanjing Drum Tower Hospital (IRB number 2016-151-01).

### Follow-up

Heart rhythm was continuously monitored after surgery. Early recurrence was defined as any episode of AF, atrial flutter or atrial tachycardia that lasted longer than 30 s in the first 3 months after surgical ablation [25]. Repeat ablation or electrical cardioversion at the follow-up time was recognized as AF recurrence. Patients had scheduled clinical visits. Twenty-four-hour Holter monitoring was routinely performed for all patients during the first 3 months after surgery. Additionally, patients received electrocardiography monitoring at local clinics at any time if they had AF-related symptoms.

### Immunofluorescence

The cells were grown on sterile coverslips, fixed with 4% paraformaldehyde and permeabilized in PBS containing 1% BSA and 0.3% Triton X-100. The cells were incubated in primary antibody (4 ?, overnight), followed by incubation in secondary antibody for 40-60 min. The primary antibodies used were as follows: anti-p21 (1:200; ab109199; Abcam), anti-p16 (1:200; ab211542; Abcam), anti-p53 (1:200; #2524; Cell Signaling), anti-αSMA (1:250; ab5694; Abcam), anti-γH2AX (1:200; #9718; Cell Signaling), and anti-troponin T (1:200; ab8295; Abcam). The secondary antibodies were Alexa Fluor488, Alexa Fluor555, Alexa Fluor594 and Alexa Fluor647 (Abcam). Coverslips were mounted in 4',6-diamidino-2-phenylindole (DAPI)-containing Vectashield and viewed using a confocal microscope (FV3000; OLYMPUS).

### Senescence-associated β-galactosidase activity assay

For in vitro studies, the cells were grown on sterile coverslips. For tissue samples, OCT-embedded frozen left atrial tissue samples (LAAs) from AF patients and controls in sinus rhythm were prepared using a commercial kit (Yeasen; 40754ES60). Briefly, the samples were fixed in 2% formaldehyde containing 0.2% glutaraldehyde for 15 min. After washing with PBS, fixed cells or tissues were stained with fresh SA-βgal Staining Solution (40 mM citric acid/Na phosphate buffer, 5 mM K_4_ [Fe(CN)_6_ ] 3H_2_O, 5 mM K_3_ [Fe(CN)_6_ ], 150 mM sodium chloride, 2 mM magnesium chloride and 1 mg ml-1 X-gal in distilled water ) and incubated at 37°C for 16 h [26]. The cells with blue color were SA-β-gal-positive. All analyses were performed in a blinded manner.

### Protein extraction and Western blot analysis

HL-1 cardiomyocytes or human tissue samples were lysed in radioimmunoprecipitation assay (RIPA) lysis buffer (Cell Signaling) supplemented with protease and phosphatase inhibitors (complete Mini and PhosSTOP; Roche) on ice for 5-15 min. After centrifugation to pellet nondissolved material, the protein concentration of each sample was determined using the bicinchoninic acid method (BCA; Thermo Fisher).

Equal amounts of protein homogenates were separated by SDS-polyacrylamide gel electrophoresis (SDS-PAGE) and transferred to nitrocellulose membranes. Membranes were blocked in blocking buffer and probed with the primary antibody (overnight at 4°C). The following primary antibodies were used: RYR2 (2446725; Millipore), p21 (ab109199; Abcam), p53 (2524; Cell Signaling), p16 (ab211542; Abcam), γH2AX (9718; Cell Signaling), CACANA1C (Cav1.2; ab84814; Abcam), SERCA2 (ab150435; Abcam), and PLB (ab219626; Abcam). The membranes were subsequently incubated with anti-mouse or anti-rabbit secondary antibodies (Abcam). Signals were detected using the enhanced chemiluminescence (ECL) detection method (Thermo Fisher) and quantified by densitometry (ImageJ). The original uncropped blots are available in the Supplementary Information section.

### Quantitative reverse transcription PCR

Total RNA was isolated from HL-1 cardiomyocytes or human samples using a TRIzol RNA isolation kit (Invitrogen). cDNA was generated according to the manufacturer’s instructions (Vazyme Biotech). Biological triplicates were analyzed using SYBR green (Vazyme Biotech) and a Roche LightCycler 480 (Roche; LC480). The comparative threshold cycles (Ct) values were normalized for GAPDH reference genes and compared with a calibrator by the 2^-ΔΔCt^ method [27]. Quantitative PCR was performed in triplicate to ensure quantitative accuracy. A primer list is available in the supplementary methods.

**Table 1:** Baseline characteristics of patients with sinus rhythm and AF.

Parameters	SR (n=12)	SAF (n=12)	LAF (n=14)	P value
Age (y)	49(42.5-55.5)	53.5(47.3-59.3)	54(50.5-60.3)	0.192
Male sex	5(41.7)	6(50)	8(57.1)	0.734
BMI (kg/m^2^)	21.8(20.35-24.3)	22.0(21.2-24.6)	24.9(23.6-27.7)	0.033*
AF history (months)	-	12.0(2.0-24.0)	114.0(93.0-240)	<0.001*
Hypertension	4(33.3)	2(16.7)	5(35.7)	0.521
Diabetes mellitus	1(8.3)	1(8.3)	0(0)	0.540
Coronary artery disease	3(25)	2(16.7)	3(21.4)	0.881
Previous stroke/TIA	0(0)	3(25)	3(21.4)	0.187
Current smoker	4(33.3)	2(16.7)	2(14.3)	0.446
Alcohol intake	3(25)	2(16.7)	2(14.3)	0.767
LAD (cm)	3.8(3.6-4.0)	5.4(5-6.2)	5.1(4.9-5.9)	<0.001*
LVEF (%)	57.0(55.0-58.0)	54.0(49.8-55.8)	51.5(47.0-55.0)	0.006*
CHA2DS2-VASc 0-1 2-3 ≥4	8(66.7) 4(33.3) 0(0)	5(41.7) 6(50) 1(8.3)	6(42.9) 7(50) 1(7.1)	0.508
ACEI/ARB				
Beta-blockers	2(16.7)	2(16.7)	7(50)	0.092
Aspirin	0(0)	1(8.3)	3(21.4)	0.198
Statins	1(8.3)	0(0)	4(28.6)	0.083
WBC (10^9^/L)	5.7(4.6-6.3)	5.1(4.3-6.3)	6.4(4.2-8.6)	0.495
BNP (pg/ml)	64.4(22.2-113.8)	375(136-590)	231.0(84.0-537.8)	0.002*
CRP (mg/L)	2.8(2.2-3.6)	3.5(2.4-4.2)	2.7(2.0-4.1)	0.53
Triglycerides (mmol/L)	1.1(0.8-1.5)	1.2 (1.0-1.6)	1.5(1.0-1.8)	0.579
Cholesterol (mmol/L)	3.9(3.0-4.4)	4.0(3.0-4.3)	3.7(3.1-4.4)	0.976
HDL (mmol/L)	1.0(0.9-1.2)	1.0(0.8-1.3)	0.9(0.8-1.2)	0.515
LDL (mmol/L)	2.2(1.6-2.6)	2.2(1.6-2.7)	2.0(1.5-2.8)	0.971
Serum creatinine	63.5(54.8-79.8)	65(52.3-76.5)	67.0(65.5-82.3)	0.305
MVD	10(83.3)	12(100)	13(92.9)	0.315
TVD	3(25)	8(66.7)	6(42.9)	0.120
AVD	1(8.3)	6(50)	3(21.4)	0.056

Continuous variables are presented as the median (interquartile range, IQR) and mean±SD, while categorical variables are presented as numbers of patients (%). Student’s t-test (normally distributed variables) or the Mann-Whitney test (non-normally distributed variables) was used for continuous variables, and the χ2 test was utilized for categorical variables. *Statistically significant value (P<0.05) AF, atrial fibrillation; SAF: short-term paroxysmal AF; LAF: long-term persistent AF; BMI, body mass index; LAD, left atrial diameter; LVEF, left ventricular ejection fraction; ACEI, angiotensin-converting enzyme inhibitors; ARB, angiotensin receptor blockers; WBC, white blood cell; BNP, B-type natriuretic peptide; CRP, C-reactive protein; HDL, high-density lipoprotein cholesterol; LDL, low-density lipoprotein cholesterol; ASD, atrial septal defect; AVD, aortic valve disease; CAD, coronary artery disease; MVD, mitral valve disease; TVD, tricuspid valve disease

### Statistical analysis

All western blotting quantifications were performed in ImageJ. Biochemical analyses were performed at least in duplicate. All statistical analyses were conducted using SPSS version 22.0 (IBM) and Prism (GraphPad Prism 7.04). Student’s t-test (normally distributed) or the Mann-Whitney test (non-normally distributed) was used for continuous variables, and the χ2 test was used for categorical variables for comparison between two groups. One-way ANOVA was performed for comparisons among three groups. Multivariate logistic regression analysis was performed to confirm the predictors of early AF recurrence. All odds ratios (ORs) are expressed with 95% confidence intervals (CIs). A receiver operating characteristic (ROC) curve was used to test the ability of the predictors, and the area under the curve (AUC) determined the predictive value. P<0.05 was considered statistically significant, and all statistical tests were two-sided.

**Figure 1.**
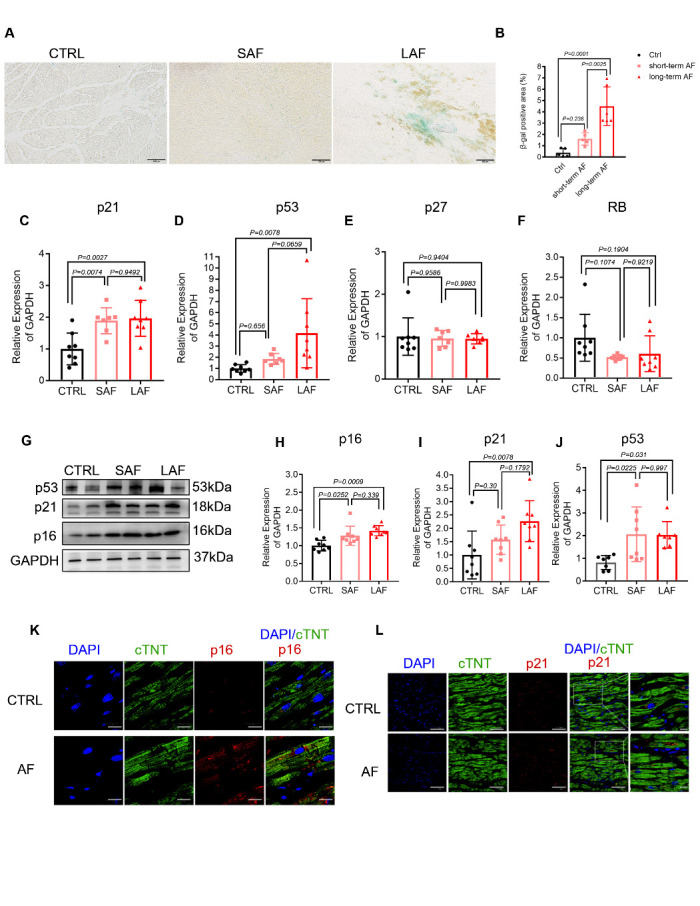
Atrial cardiomyocyte senescence accumulates in LAAs with AF. (A-B) Representative images and quantification of each group of LAAs staining positive for SA-ß-gal (blue). All the samples were fresh-frozen, OCT-embedded LAA sections (bar = 100 μm; n = 3 per group). (C-F) RT-qPCR analysis of senescence-associated genes in human LAAs in each group. GAPDH was used as a housekeeping gene (n=7-8 in each group). (G-J) Western blot analysis of senescence-associated protein levels in human LAAs in each group. GAPDH was used as a loading control (n=7-8 in each group). (K) Immunofluorescence co-staining for cTNT (green) and p16 (red) in LAA sections from patients with SR or AF. The nuclei were stained with DAPI (n = 2 per group; bar=20 μm). (I) Immuno-fluorescence costaining for cTNT (green) and p21 (red) in LAA sections from patients with SR or AF. The nuclei were stained with DAPI (n = 2 per group; bar = 50 μm). Colocalizing foci are amplified in the right-hand panels (bar = 20 μm). Comparisons were performed using one-way ANOVA.

## RESULTS

### Patient characteristics

The baseline characteristics of individuals with sinus rhythm and AF used for RT-qPCR, Western blot and immunofluorescence analyses are shown in Table 1.

Twenty-six patients, including 12 with SAF and 14 with LAF, were compared with 12 matched patients with sinus rhythm.

No statistically significant differences were found with respect to age, sex, cardiovascular risk factors, CHA2DS2-VASc scores or drug treatment (β-blockers, statins, angiotensin-converting enzyme (ACE) inhibitors, or AT1 blockers) among the 3 groups, indicating that the 3 groups were homogeneous.

Significant differences were found in the body mass index (BMI), AF duration history, left atrial diameter (LAD), left ventricular ejection fraction (LVEF) and BNP among the 3 groups of patients included in this study.

### Atrial cardiomyocyte senescence accumulates in LAAs with AF

To clarify the link between cell senescence and AF, we measured SA-β-gal activity and expression of the senescence markers p53, p21 and p16 in left atrial appendages (LAAs) from AF and sinus rhythm patients. The LAF group was associated with higher expression of SA-β-gal-positive cells than the sinus rhythm and SAF groups (Fig. 1A-B). RT-qPCR and Western blotting (WB) analyses indicated that expression of p53, p21 and p16 was significantly upregulated in LAF patients compared with sinus rhythm controls (Fig. 1C-J). We also detected increased expression of p21 and p16 in the SAF group, although the number of SA-β-gal-positive cells was low, suggesting an early tendency toward cell senescence in those patients (Fig. 1 G-J). Additionally, we performed immunofluorescence to identify specific types of senescent cells in LAAs. Most of the senescent cells (p21- and p16-positive) expressed troponin T (cTNT), a classical cardiomyocyte marker [28,29] (Fig. 1K-L and Supplementary Fig. 1A-B). Together, these data support the notion that atrial cardiomyocyte senescence increases with AF.

**Table 2:** Characteristics of AF patients after 3 months following the maze procedure.

Parameters	Total n=120	SR maintenance n=45	AF recurrence n=75	P
Age (y)	59(53-66)	57(53-67)	59(54-66)	0.425
Sex male female	58(48) 62(52)	18(40) 27(60)	40(53) 35(47)	0.157
BMI (kg/m^2^)	23.9(21.7-26.4)	23.8(21.5-25.8)	24(21.7-26.8)	0.404
AF duration history (m)	24 (3-120)	6(1-90)	36(12-120)	0.018*
Hypertension	44(37)	16(36)	28(37)	0.845
Diabetes	9(7.5)	4(9)	5(7)	0.655
CAD	18 (15)	8(18)	10(13)	0.509
Heart failure	45(37.5)	17(38)	28(37)	0.237
TIA/Stroke	9 (7.5)	2(4.4)	7(9.3)	0.325
Current smoker	13(11)	4(9)	9(12)	0.596
Alcohol intake	8(7)	3(7)	5(7)	1.00
LAD (cm)	5.2 (4.6-6.0)	5.1(4.4-5.5)	5.4(4.8-6.2)	0.015*
LVEF (%)	54 (47-58)	55(48-58)	54(46-58)	0.650
CHA2DS2-VASc score	2(2-3)	2(2-3)	2(2-3)	0.95
WBC (10^9/L)	5.6(4.6-6.6)	5.7(4.9-6.6)	5.4(4.3-6.7)	0.78
BNP (pg/ml)	235(143-450)	270(152-595)	213(136-412)	0.189
CRP (mg/L)	3.7(2.6-4.9)	2.8(2.1-4.3)	4.0(3.1-5.4)	0.001*
Triglycerides (mmol/L)	1.1(0.8-1.5)	1.3(1.0-1.6)	1.0(0.8-1.4)	0.002*
Cholesterol (mmol/L)	3.6(3.2-4.1)	3.8(3.5-4.3)	3.5(2.9-4.0)	0.002*
*CDKN1A* relative expression	0.012 (0.006-0.02)	0.009 (0.005-0.014)	0.014 (0.009-0.023)	<0.001*

* Statistically significant value (P<0.05) AF, atrial fibrillation; BMI, body mass index; CAD, coronary artery disease; LAD, left atrial diameter; LVEF, left ventricular ejection function; WBC, white blood cell; BNP, B-type natriuretic peptide; CRP, C-reactive protein; CDKN1A, cyclin-dependent kinase inhibitor 1A.

### AF recurrence is closely linked to the premature senescence burden

**Figure 2.**
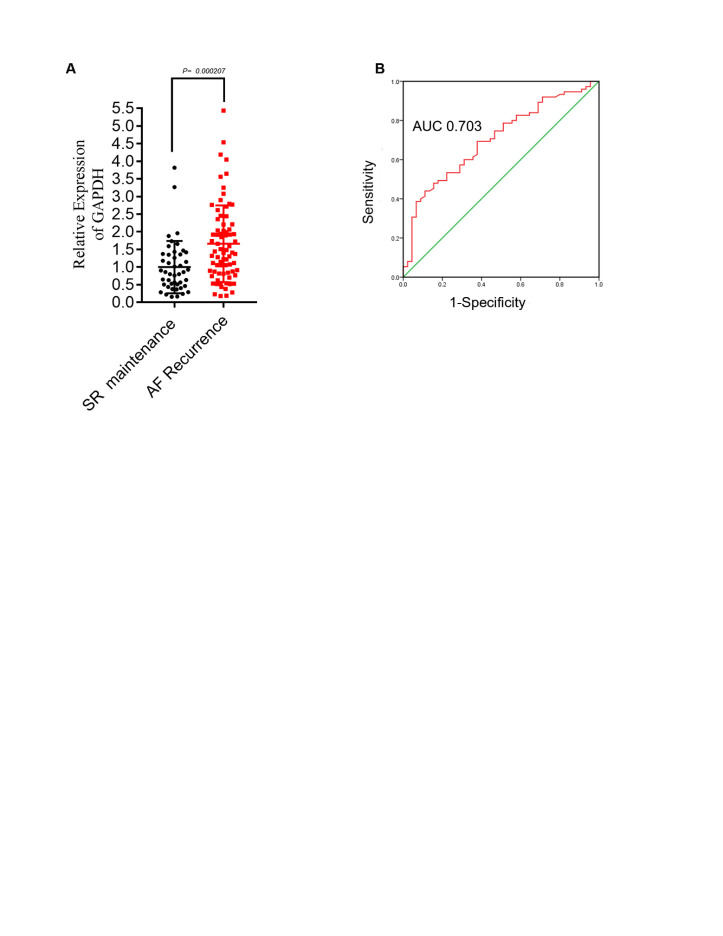
AF recurrence is closely related to the premature senescence burden. (A) Comparison of p21 mRNA (CDKN1A) expression due to AF recurrence and SR maintenance. Comparisons were performed using the Mann-Whitney test. P=0.000207 for SR maintenance (n=45) vs. AF recurrence (n=75). (B) Receiver operating characteristic curve of p21 for predicting AF recurrence after the maze operation (AUC, area under the curve; CI, confidence interval).

Recently, it was reported that cell senescence is strongly related to AF progression [18]. We hypothesized that senescence may have predictive value in AF recurrence. In total, 120 patients with consecutive persistent valvular AF who required a concomitant radiofrequency (RF) maze procedure and mitral valve surgery were enrolled, and LAAs were obtained from those patients undergoing cardiac surgery. All patients completed a 3-month follow-up after surgery. Expression of p21 (*CDKN1A*) in appendage lysates was evaluated by RT-qPCR relative to sinus rhythm maintenance and AF recurrence. The patients with early AF recurrence had higher *CDKN1A* levels than those with sinus rhythm maintenance (Table 2 and Fig. 2A). In multivariate analysis, *CDKN1A* levels (OR: 2.97; 95% CI: 1.65-5.34; P<0.001) were found to be significantly independent predictors of AF early recurrence (AUC=0.703) (Table 3 and Fig. 2B). These results indicate that AF recurrence is closely related to senescence burden, as determined by p21 expression.

### TP induces HL-1 atrial cardiomyocytes senescence in vitro

**Figure 3.**
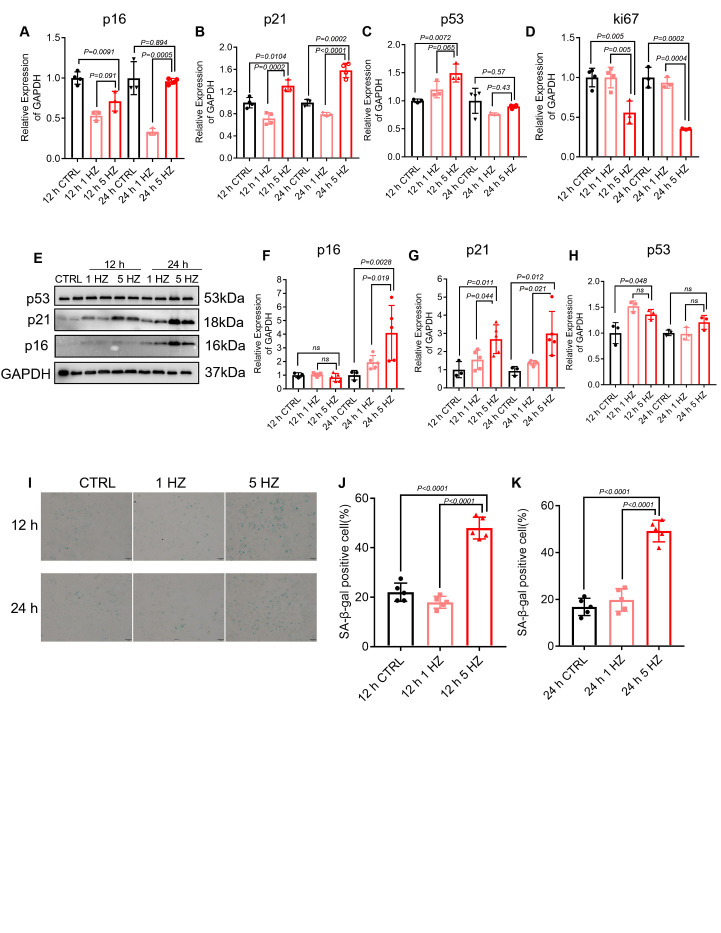
TP induces HL-1 atrial cardiomyocytes senescence in vitro. (A-D) Senescence-associated mRNA expression was determined by RT-qPCR analysis in control (0 HZ; CTRL), normal-paced (1 HZ) and tachypaced (TP) (5 HZ) HL-1 cardiomyocytes for the indicated durations (n=3-4). (E-H) Western blot and corresponding quantification analyses of senescence-associated protein expression in control (0 HZ; CTRL), normal-paced (1 HZ) and TP (5 HZ) HL-1 cardiomyocytes for the indicated durations. GAPDH was used as a loading control (n=3-5). (I-K) SA-β-Gal staining in control nonpaced (0 HZ, CTRL), normal-paced (1 HZ) and TP (5 HZ) HL-1 cardiomyocytes for the indicated durations (blue: SA-β-Gal; scale bar 1 mm) (n=3). Comparisons were performed using one-way ANOVA for three groups and Student’s t-test for two groups.

Tachypaced HL-1 atrial cardiomyocytes have been used as experimental model systems for AF[23]. To determine whether TP could induce cellular senescence in vitro, HL-1 atrial cardiomyocytes were subjected to no pacing (0 HZ), normal pacing (1 HZ) or tachypacing (TP, 5 HZ), followed by the examination of markers of cellular senescence. Twelve hours of TP significantly increased p21 expression and SA-β-gal activity while decreasing Ki-67 expression compared with no pacing and normal pacing (Fig. 3A-K). Moreover, 24 h of TP significantly increased p21 and p16 expression and SA-β-gal activity and decreased Ki-67 expression compared with no pacing and normal pacing (Fig. 3A-K). A gradual increase in p53 levels was observed after 12 and 24 h of TP, although no significant differences were found between groups.

**Table 3:** Univariate and multivariate analyses of risk factors for early recurrence after the maze procedure.

Parameters	Univariate model			Multivariate model		
	OR	95% CI	P	OR	95% CI	P
Age	1.01	0.97-1.06	0.453			
AF duration history	1.00	0.99-1.00	0.186			
LAD	1.85	1.18-2.89	0.007	1.85	1.11-3.10	0.019*
CRP	1.34	1.06-1.71	0.017	1.38	1.06-1.78	0.015*
Triglycerides	0.27	0.12-0.62	0.002	0.73	0.27-2.02	0.545
Cholesterol	0.45	0.26-0.77	0.004	0.37	0.18-0.76	0.007*
*CDKN1A* gene expression	2.43	1.42-4.16	0.001	2.97	1.65-5.34	<0.001*

* Statistically significant value (P<0.05) OR, odds ratio; CI, confidence interval; LAD, left atrial diameter; CRP, C-reactive protein; CDKN1A, cyclin-dependent kinase inhibitor 1A.

Abundant data have shown that atrial myocytes express all components of the renin-angiotensin system (RAS) and angiotensin II (AngII), which is produced by atrial myocytes and plays important roles during atrial structural and electrical remodeling in AF [23, 30]. Additionally, inhibition of the cardiac RAS by ACE inhibitors and/or AngII receptor blockers (ARBs) provides a therapeutic option for AF [31-33]. Considering the elevation of AngII in atrial tissues, we treated HL-1 cells with AngII for 48 h and subjected them to no pacing (0 HZ, CTRL), normal pacing (1 HZ), or TP (5 HZ), followed by the evaluation of markers of cellular senescence. As illustrated in Supplementary Figure S2, Ang II alone did not affect expression of p53, p21 or p16 (Supplementary Fig. 2J-M). Ang II combined with TP for 12 h markedly increased cellular expression of p21 and SA-β-gal activity compared with no pacing and normal pacing (Supplementary Fig. 2A-I). Moreover, AngII combined with tachypacing for 24 h significantly increased p53, p21, and p16 expression and SA-β-gal activity compared with no pacing and normal pacing (Fig. S2J-M). These observations indicate that TP induces HL-1 cell senescence.

**Figure 4.**
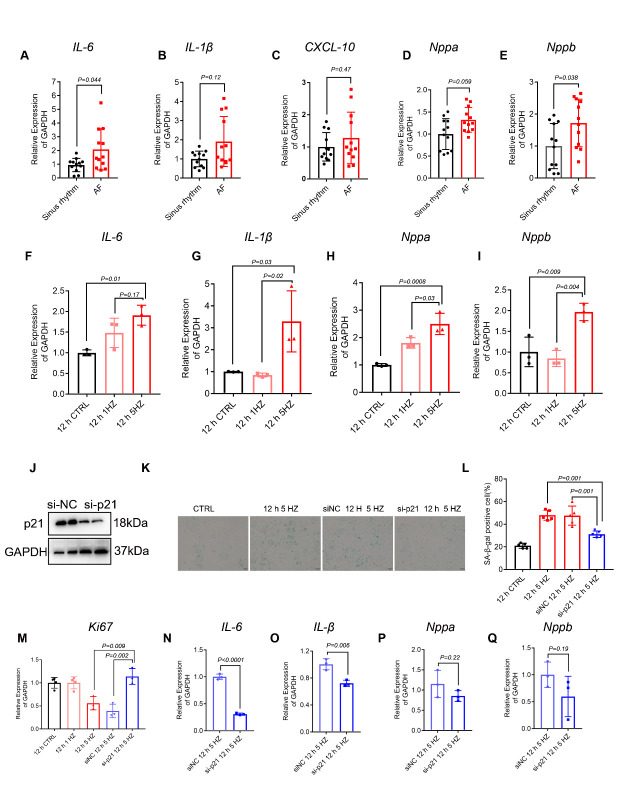
Suppression of TP induced cell senescence reduced SASP elevation. (A-E) The mRNA expression of IL-1β, IL-6, CXCL10, Nppa, and Nppb was determined by qPCR analysis in LAAs (n=12). (F-I) The mRNA expression of IL-1β, IL-6, Nppa, and Nppb was determined by qPCR analysis in control nonpaced (0 HZ; CTRL), normal-paced (1 HZ) and tachypaced (TP) (5 HZ) HL-1 cardiomyocytes for the indicated durations (n=3). (J) Protein expression of p21 after treatment with siNC and si-p21 (n=3). (K-M) SA-β-Gal staining and quantification analyses of cells after treatment with siNC TP for 12 h or si-p21 TP for 12 h. (n=3). *Ki67* mRNA expression was also analyzed after treatment with siNC TP for 12 h or sip21 TP for 12 h (n=3). (N-Q) IL-6, IL-1β, Nppa and Nppb mRNA expression was analyzed after treatment with siNC TP for 12 h or sip21 TP for 12 h (n=3). Comparisons were performed using one-way ANOVA for three groups and Student’s t-test for two groups.

**Figure 5.**
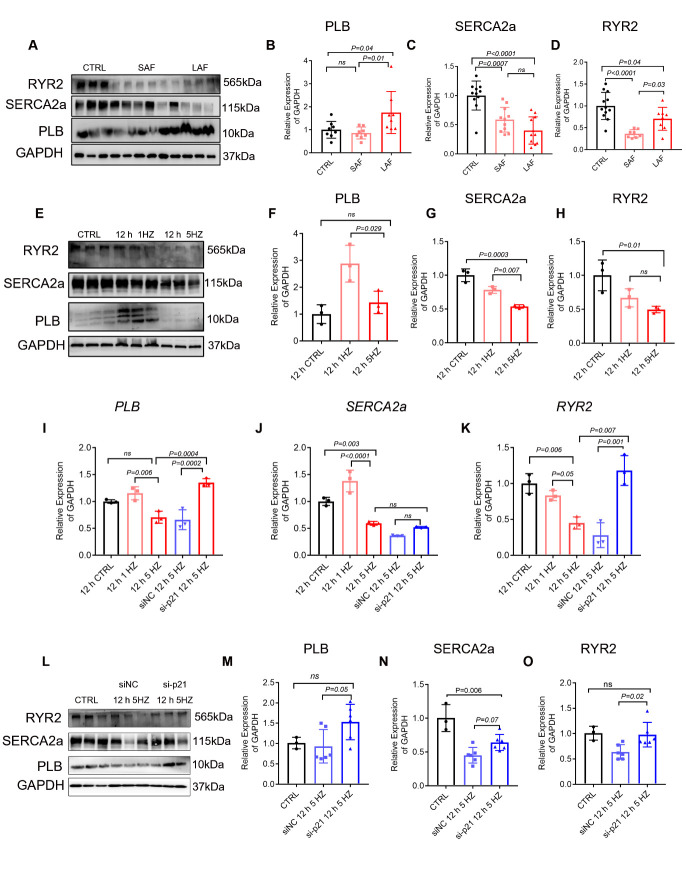
TP-induced senescence partly modulates sarcoplasmic reticulum-related proteins. (A-D) Western blot and quantification analyses of PLB, SERCA2a and RYR2 expression in human samples (n=8-12). (E-H) The protein expression of PLB, SERCA2a and RYR2 was determined by Western blot analysis in control nonpaced (0 HZ; CTRL), normal-paced (1 HZ) and TP (5 HZ) cells for 12 h. GAPDH was used as a loading control (n=3). (I-K) mRNA expression of PLB, SERCA2a and RYR2 after treatment with siNC TP or si-p21 TP for 12 hours (n=3). (L-O) Protein expression and quantification analyses of PLB, SERCA2a and RYR2 after treatment with CTRL, siNC TP or si-p21 TP for 12 hours (n=3-6). Comparisons were performed using one-way ANOVA for three groups and Student’s t-test for two groups.

### Suppression of TP induced cell senescence reduced SASP elevation

The SASP is the main hallmark of cellular senescence[9]. SASP signaling involves a variety of biologically active proinflammatory cytokines, chemokines, and remodeling factors that induce malignant phenotypes in nearby cells by modulating inflammatory processes and tissue microenvironments[34]. We analyzed typical SASP expression in the LAAs of AF and SR patients, showing that IL-6 and NPBB levels were significantly increased in AF patients compared with SR patients (Figure 4A-E). To identify the SASP factors secreted by TP-induced senescent HL-1 cells, we measured the expression of genes involved in SASP. According to confirmatory RT-qPCR, TP significantly increased transcriptional expression of inflammatory and prohypertrophic markers associated with SASP, such as Il-1β, Il-6, Nppa, and Nppb (Figure 4F-I). Furthermore, we knocked down p21 with siRNA in HL-1 cardiomyocytes and then subjected the cells to 5 HZ TP for 12 h, and expression of p21 at the mRNA and protein levels was confirmed after siRNA knockdown (Fig. 4J, Supplementary Fig. 3A-D). p21 knockdown with siRNA significantly decreased TP-induced HL-1 cell senescence (Fig. 4K-M). Changes in Il-1β and Il-6 at the mRNA level were also observed in si-p21 versus siNC following TP (Fig. 4N-Q). Taken together, our results show that TP induced senescence accompanied by elevated expression of proinflammatory and hypertrophic markers, suggesting that atrial cardiomyocyte senescence may be involved in structural remodeling in AF.

**Figure 6.**
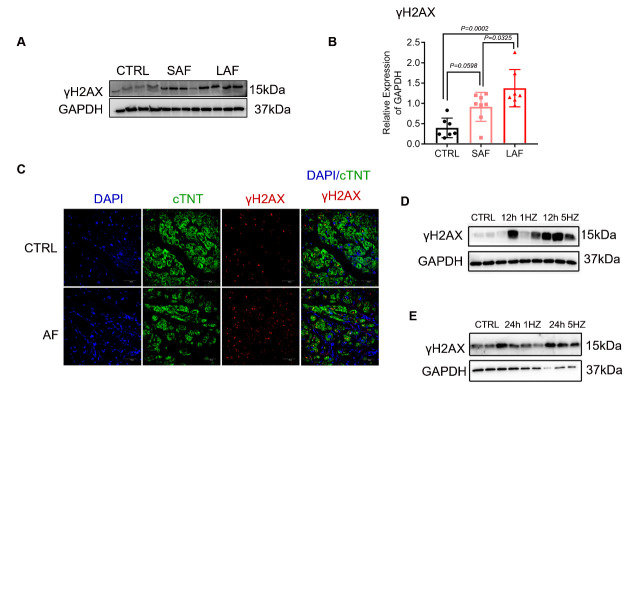
DNA damage-related foci formation increased in senescent atrial cardiomyocytes and in AF. (A-B) Western blot analysis of the DNA damage-associated protein γH2AX in human LAAs in each group. GAPDH was used as a loading control (n=7-8). (C) Immunofluorescence staining for cTNT (green) and γH2AX (red) in LAA sections from patients with SR or AF (n=2; bar = 50 μm). (D-E) Western blot analysis of γH2AX protein expression in control nonpaced (0 Hz; CTRL), normal-paced (1 Hz) and TP (5 Hz) HL-1 cardiomyocytes for the indicated durations (n=3). Comparisons were performed using one-way ANOVA for three groups.

### TP-induced senescence partly modulates sarcoplasmic reticulum-related proteins

Sarcoplasmic reticulum (SR) Ca^2+^ homeostasis is crucial for the contraction and relaxation of cardiomyocytes, and it is becoming more evident that the maintenance of SR Ca^2+^ homeostasis is highly dependent on the coordinated action of SR-related proteins [35]. Multiple lines of evidence indicate that the discoordination of several SR-related proteins, including SERCA, RyR2 and PLB, contributes to further deterioration of cardiac performance among AF patients [19, 36]. We analyzed the expression of the major SR Ca^2+^ channel proteins RyR2, SERCA2a and PLB in the LAAs of AF and SR patients, showing that the SERCA2a and RYR2 protein levels were significantly decreased and PLB was increased in AF patients compared with SR patients (Fig. 5A-D). Furthermore, we explored the link between cellular senescence and SR-related proteins and found that mRNA and protein expression of PLB, SERCA2A and RYR2 decreased after 12 h of TP compared with normal pacing (Fig. 5E-H, Supplementary Fig. 4A-D). SERCA2Aa and RYR2 protein expression also decreased after 24 h of TP compared with normal pacing; however, surprisingly, PLB expression was increased at this time point (Supplementary Fig. 4A-H). The concurrent senescence-related expression pattern of several SR proteins indicated that atrial cardiomyocyte senescence is closely related to SR Ca^2+^ proteins.

To further substantiate our hypothesis that TP-induced atrial cardiomyocyte senescence caused alteration of SR proteins, we knocked down p21 with siRNA in HL-1 cardiomyocytes and then subjected the cells to 5-HZ TP for 12 h. Changes in SERCA2a, PLB and RYR2 at the mRNA and protein levels were also observed in si-p21 versus siNC following TP (Fig. 5I-O). Suppression of HL-1 cell senescence restored SERCA2a and RYR2 level. These results suggest that TP-induced premature senescence partly modulates SR-related proteins.

### DNA damage-related foci formation increased in senescent atrial cardiomyocytes and in AF

Recent studies have shown that AF correlates with DNA damage, and abundant data show that DNA damage is the major trigger of cell senescence [9, 37,38]. To establish the link between DNA damage and atrial cardiomyocyte senescence in AF, we measured phosphorylation of the Ser-139 residue of the histone variant H2AX (γH2AX), an established marker of DNA damage [39]. In accordance with the literature, the γH2AX levels in atrial cardiomyocytes were markedly increased in patients with AF compared with SR patients (Fig. 6A-C). Additionally, a significant increase in γH2AX levels was observed following TP (Fig. 6D-E and Supplementary Figure 5A-B). DNA damage-related foci formation increased in senescent atrial cardiomyocytes and in LAAs, which indicated close links between AF, DNA damage and cellular senescence and further clarified atrial cardiomyocyte senescence in AF.

## DISCUSSION

To our knowledge, this study is the first to report the cellular senescence of atrial cardiomyocytes in patients with atrial fibrillation.

We first characterized SA-βgal activity and the expression of the senescence-associated molecular markers p53/p21 and p16 in LAAs from control and AF subjects. Increased SA-βgal activity and levels of p53, p21, and p16 in the atrium were observed in AF subjects aged younger than 60 years compared with control subjects. Immunofluorescence assays showed that atrial cardiomyocyte senescence increases with AF. In conjunction with the literature and considering the critical role of p21 in stress-induced premature senescence, our data demonstrate that p21 expression is an independent risk factor for early AF recurrence after surgery, suggesting that premature cell senescence likely contributes to AF progression. Our in vitro data also showed that TP significantly induces HL-1 cell senescence and SASP components. Chronic inflammation may also be derived in part from senescent cells, and elevated IL-1β and IL-6 are associated with age-related disease [40,41]. our results showed that IL-1β and IL-6 upregulated in TP-induced senescent cells. Suppression of p21 by siRNA reduced TP induced cell senescence and IL-1β and IL-6 elevation. Furthermore, TP-induced HL-1 cell senescence is accompanied by alterations of several SR proteins, consistent with human LAAs, and this induction is partly inhibited by suppression of p21 expression, suggesting that suppression of atrial cardiomyocyte senescence may help to improve atrial remodeling in AF. Collectively, these findings likely account for the finding that cell senescence is a risk factor for AF recurrence in patients. Our study supports the notion that AF significantly contributes to the acceleration of atrial cardiomyocyte senescence, leading to AF progression. Finally, we showed that molecular marker of senescence-associated DNA damage increased in AF individuals and experimental AF. These observations may account for the observed AF-induced atrial cardiomyocyte senescence, thus contributing to deleterious atrial remodeling during AF progression, and may favor a progressive decline in atrial function, bringing challenges to AF treatment.

Although a close link between aging and AF has been demonstrated in epidemiological studies, little is known regarding the cellular and molecular alterations of atrial tissues during aging. Cellular senescence is characterized by biochemical events that lose certain cellular functions and contribute to accelerated organ aging [42]. Premature cellular senescence and tissue aging can contribute significantly to the complexity of AF pathogenesis by different means [7, 18, 19]. Recently, the key roles of senescence have attracted attention in cardiovascular diseases, including AF [16, 43]. Additionally, Passos *et al* found that length-independent telomere damage induces cardiomyocyte senescence, and that the clearance of senescent cells significantly attenuates age-related cardiac dysfunction [16]. Laurence *et al* found that AF progression is closely related to the human atrial senescence burden [18]. Additionally, studies have shown that senescence markers, such as SA-β-gal activity and p16, correlate positively with the extent of atrial fibrosis [20, 44]. Given these findings, we propose that AF may induce premature cell senescence independent of age and that premature cell senescence may be one of the mechanisms underlying AF progression. Our results indicated that cellular senescence plays an essential role in AF progression.

It has been well established that p53 modulates senescence by increasing p21 expression [10]. As a strong inducer of cell senescence, p16 is also involved in cardiomyocyte senescence [16, 45]. In the present study, total p53 was not markedly increased in TP, although p21 and/or p16 was upregulated. One reason for this result is that the intensity, duration or environmental cues of the inciting stimulus in vitro may differ in clinical AF, which is a multicellular and far more complicated disease than experimental AF. Another reason is that p53 is involved in various signaling pathways in addition to cellular senescence, and acetylated p53 levels may better reflect cardiomyocyte senescence than total p53 [46].

Cells undergo senescence in response to identical stimuli. A recent study showed that persistent DNA damage increases with age in cardiomyocytes and can be induced by mitochondrial dysfunction [16, 47, 48]. Zhang *et al* also found that AF is associated with DNA damage, which subsequently induces atrial cardiomyocyte contractile dysfunction and disease progression [37]. In the present study, we examined γH2AX, a marker of DNA damage, in experimental AF and validated findings in clinical AF. We showed that DNA damage increased in senescent atrial cardiomyocytes and in AF. Our results highlight that the complex pathological state of AF underlies cell senescence, launching the search for new therapeutic approaches for antiaging therapy. At the same time, a deep understanding of the mechanistic pathways linking senescence to atrial tissue remodeling in different clinical scenarios in AF is necessary for developing specific therapeutic strategies for the primary or secondary prevention of this disease.

## Limitations

First, despite efforts to match our study patients, we could not exclude potential sources of bias. Second, valvular AF does not necessarily reflect mechanisms in nonsurgical AF of different etiologies. Thus, the results may not be generalizable to all AF populations. Third, definitive mechanisms of cellular senescence and its impact on atrial cardiomyocyte electrophysiology involving AF is undiscussed and remain largely unknown. Finally, whether eliminating senescent cells or antiaging therapy can reduce AF recurrence and help to improve AF outcomes needs to be further evaluated.

## Conclusions

AF underlies cardiomyocyte senescence and contributes to deleterious atrial remodeling during disease progression. This finding should pave the way for the development of new therapeutic approaches for antiaging therapy for AF.

## Supplementary Materials

The Supplementary data can be found online at: www.aginganddisease.org/EN/10.14336/AD.2021.0619.


