# Supplementary material for: Atrial Fibrillation Underlies Cardiomyocyte Senescence and Contributes to Deleterious Atrial Remodeling during Disease Progression
Source: Aging Dis. 2022 Feb 1;13(1):298–312. doi: 10.14336/AD.2021.0619 (PMC8782549; doi:10.14336/AD.2021.0619)
Supplement: Supplementary file 1 [file AD-13-1-298-s.pdf]

SUPPLEMENTARY DATA

Atrial Fibrillation Underlies Cardiomyocyte Senescence and Contributes to Deleterious Atrial Remodeling during Disease Progression

Ailiya Adili[#], Xiyu Zhu[#], Hailong Cao, Xinlong Tang, Yali Wang, Junxia Wang, Jian Shi, Qing Zhou[*], Dongjin Wang[*]

© 2021. Liu Y et al. Published online at http://www.aginganddisease.org/EN/10.14336/AD.2021.0502

SUPPLEMENTARY DATA

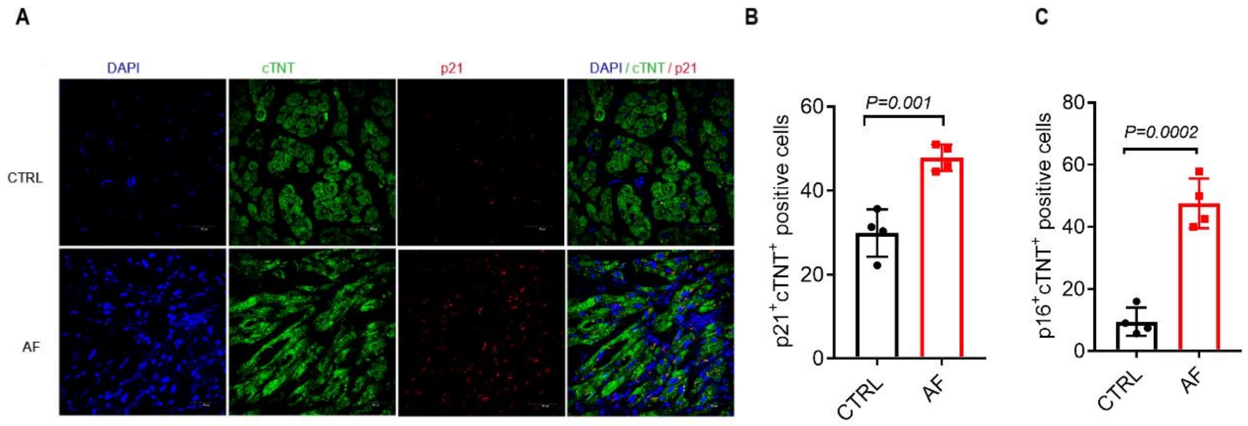

Supplementary Figure 1. Atrial cardiomyocyte senescence accumulated in LAAs with AF. (A) Immunofluorescence co-staining for cTNT (green) and p21 (red) in LAA sections from patients with SR or AF (bar = 50 μm). **(B)** Graph showing the percentage of p21-cTNT positive cells and p16-cTNT in total cell of LAAs.

© 2021. Liu Y et al. Published online at http://www.aginganddisease.org/EN/10.14336/AD.2021.0502

SUPPLEMENTARY DATA

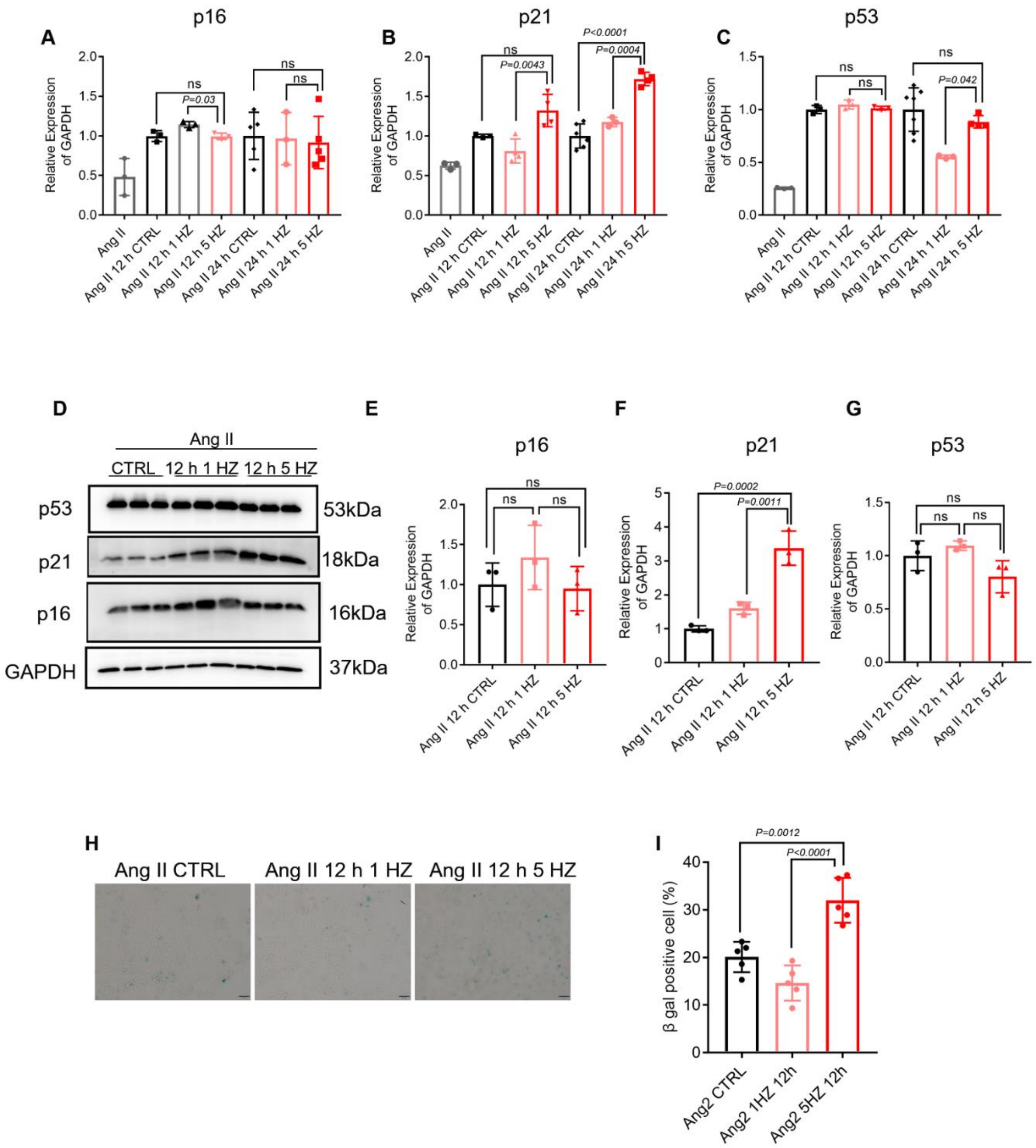

© 2021. Liu Y et al. Published online at http://www.aginganddisease.org/EN/10.14336/AD.2021.0502

SUPPLEMENTARY DATA

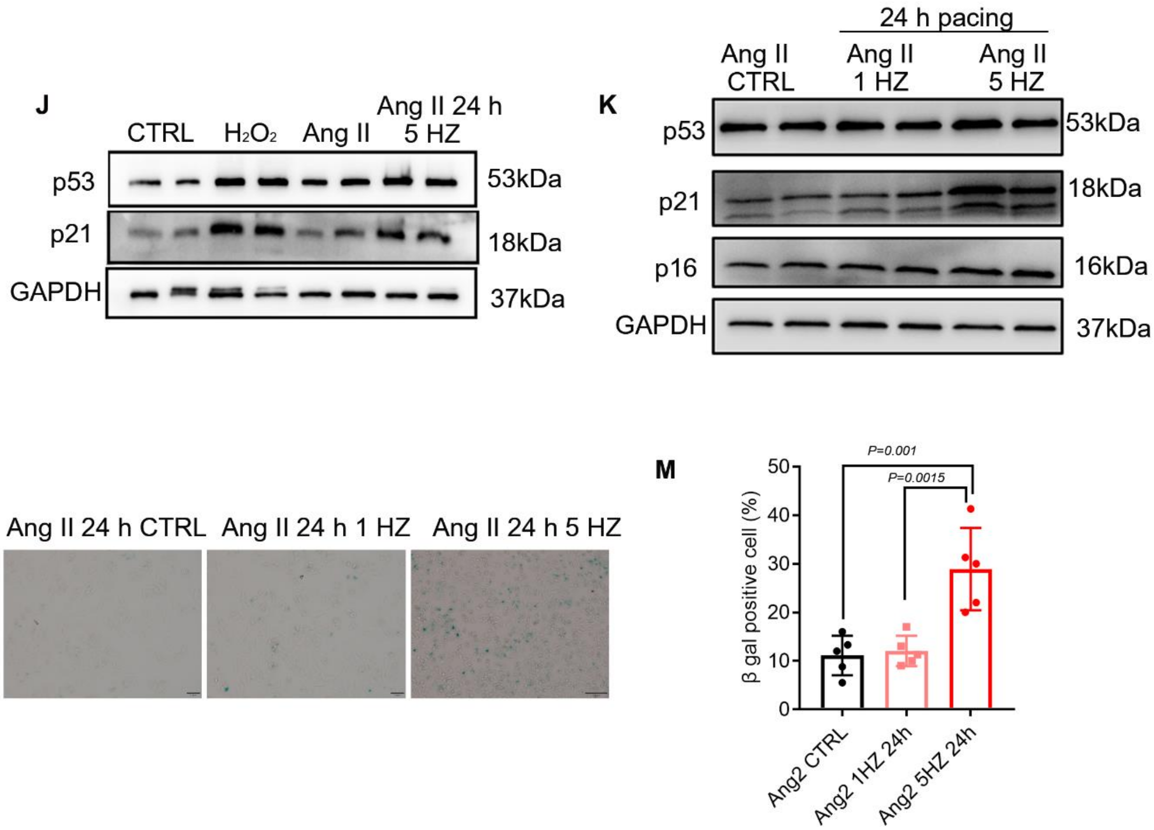

Supplementary Figure 2.TP induces HL-1 atrial cardiomyocytes senescence in vitro. (A-C) Senescence-associated mRNA expression was determined by qPCR analysis of Ang II combined with nonpaced (Ang II CTRL), normal-paced (Ang II 1 HZ) and tachypaced (TP) (Ang II 5 HZ) HL-1 cardiomyocytes for the indicated durations (n=3-5). **(D-G)** Western blot and quantification analyses of senescence-associated protein expression of Ang II combined with nonpaced (Ang II CTRL), normal-paced (Ang II 1 HZ and TP (Ang II 5 HZ) HL-1 cardiomyocytes for 12 h. GAPDH was used as a loading control (n=3). **(H-I)** SA-β-Gal staining of Ang II combined with nonpaced (Ang II CTRL), normal-paced (Ang II 1 HZ) and tachypaced (TP) (Ang II 5 HZ) HL-1 cardiomyocytes for 12 h (blue: SA-β-Gal; scale bar=1 mm) (n=3).**(J)** HL-1 cardiomyocytes were subjected to the following treatments: control, 100 μM hydrogen peroxide (H2O2) for 1 h, Ang II for 48 h and Ang II for 48 h, followed by TP (5 HZ) for 24 h. Next, senescence-associated protein expression was assessed and quantified in each group. GAPDH was used as a loading control (n=2). **(K)** Western blot analysis of senescence-associated protein expression of Ang II combined with nonpaced (Ang II CTRL), normal-paced (Ang II 1 HZ) and TP (Ang II 5 HZ) HL-1 cardiomyocytes for 24 h. GAPDH was used as a loading control (n=2). **(L-M)** SA-β-Gal staining of Ang II for 48 h combined with nonpaced (0 HZ), normal-paced (1 HZ) and tachypaced (TP) (5 HZ) HL-1 cardiomyocytes for 24 h (blue: SA-β-Gal, scale bar 1 mm) (n=3).

© 2021. Liu Y et al. Published online at http://www.aginganddisease.org/EN/10.14336/AD.2021.0502

SUPPLEMENTARY DATA

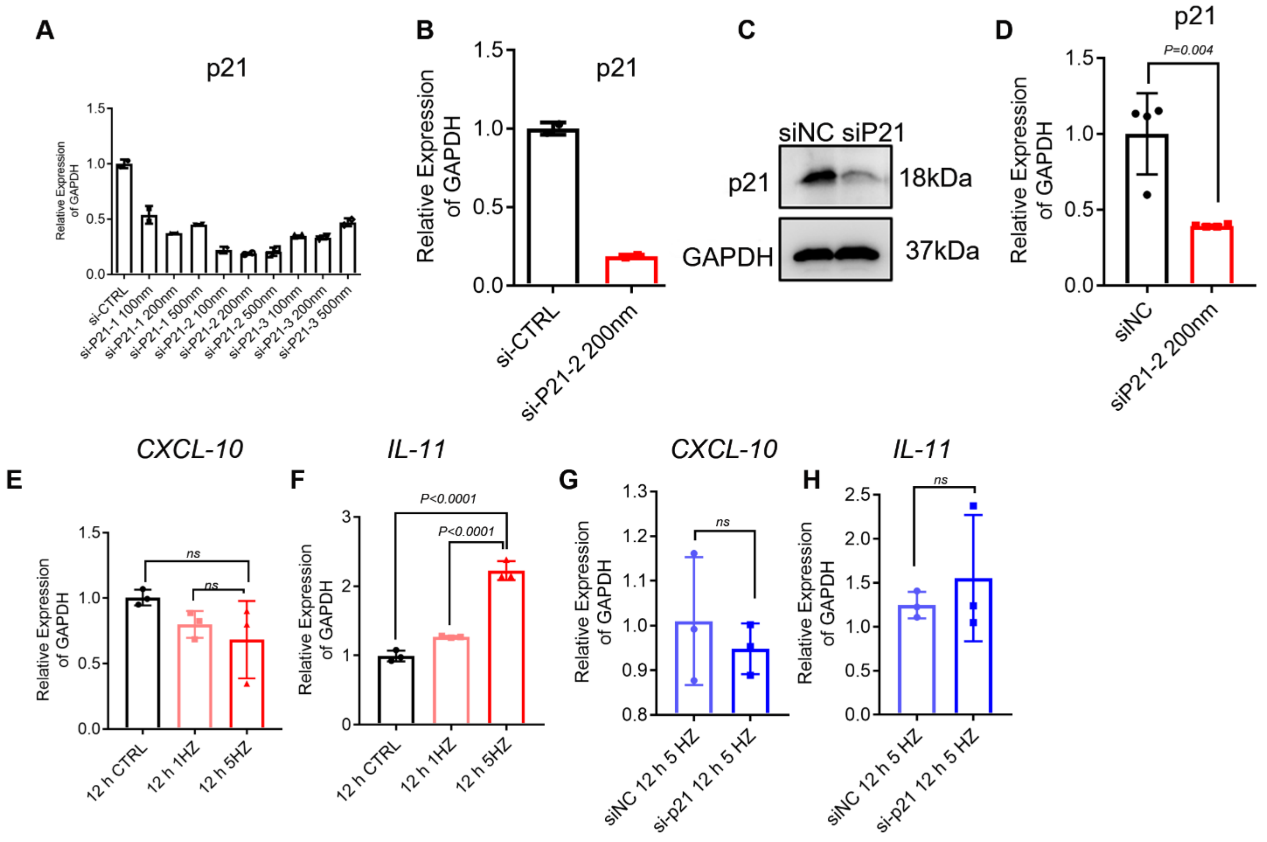

Supplementary Figure 3. Suppression of TP induced cell senescence reduced SASP elevation. (A-D) mRNA expression of p21 analyzed after treatment with siNC and sip21. **(E-H)** The mRNA expression of IL-11 and CXCL-10 was determined by qPCR analysis in control nonpaced (0 HZ; CTRL), normal-paced (1 HZ) and TP (5 HZ) HL-1 cardiomyocytes for the indicated durations (n=3).

© 2021. Liu Y et al. Published online at http://www.aginganddisease.org/EN/10.14336/AD.2021.0502

SUPPLEMENTARY DATA

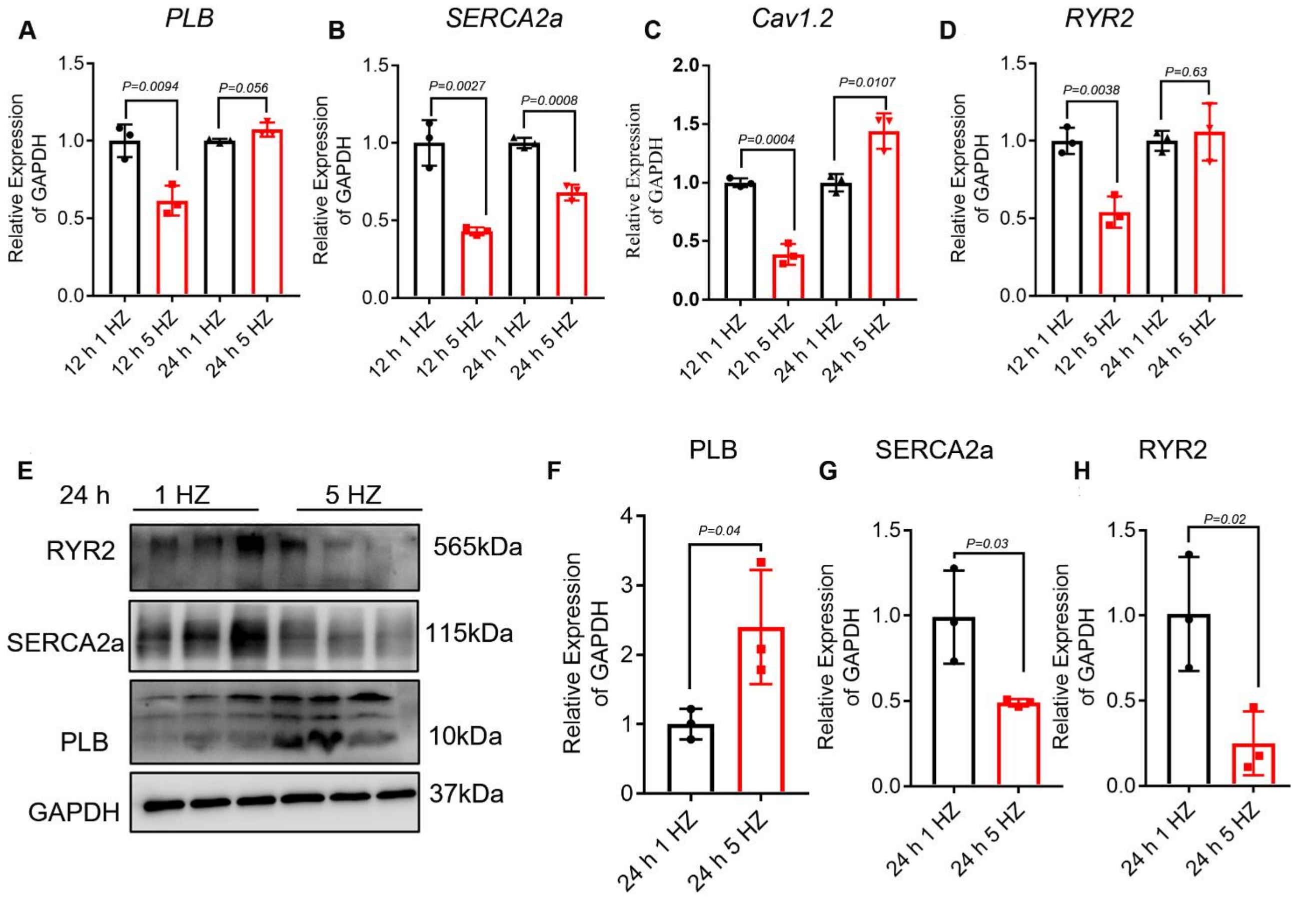

Supplementary Figure 4. TP-induced senescence partly modulates sarcoplasmic reticulum-related proteins. (A-D) The mRNA expression of PLB, SERCA2a, Cav1.2 and RYR2 was determined by RT-qPCR analysis in normal-paced (1 HZ) and TP (5 HZ) HL-1 cardiomyocytes for the indicated durations (n=3). **(E-H)** The protein expression of PLB, SERCA2a and RYR2 was determined by Western blot analysis in normal-paced (1 HZ) and TP-treated (5 HZ) cells for 24 h. GAPDH was used as a loading control (n=3).

© 2021. Liu Y et al. Published online at http://www.aginganddisease.org/EN/10.14336/AD.2021.0502

SUPPLEMENTARY DATA

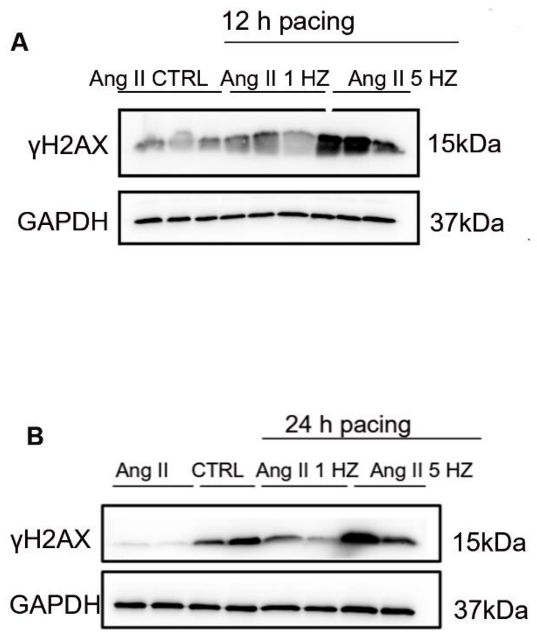

Supplementary Figure 5. DNA damage-related foci formation increased in senescent atrial cardiomyocytes and in AF. (A-B) Western blot analysis of γH2AX protein expression in Ang II combined with nonpaced, normal-paced and TP HL-1 cardiomyocytes for the indicated durations. GAPDH was used as a loading control (n=2).

Primers used in this study (H: human; M: mouse)

Primers	Sequence
H-CDKN1A F	TGTCCGTCAGAACCCATGC
H-CDKN1A R	AAAGTCGAAGTTCCATCGCTC
H-P53 F	TGCTCAAGACTGGCGCTAAA
H-P53 R	AGTCTGGCCAATCCAGGGAA
H-PARP1 F	CCCTAAAGGCTCAGAACGACC
H-PARP1 R	AGGAGGGCACCGAACACC
H-P27 F	GCAGCTTGCCCGAGTTCT
H-P27 R	AGAAGAATCGTCGGTTGCAGG
H-RB F	TTGTAACGGGAGTCGGGAGA
H-RB R	CTCAAGCCTGACGAGAGGCAG
H-GAPDH F	GGAGCGAGATCCCTCCAAAAT
H-GAPDH R	GGCTGTTGTCATACTTCTCATGG

© 2021. Liu Y et al. Published online at http://www.aginganddisease.org/EN/10.14336/AD.2021.0502

M-CDKN1A F	CCTGGTGATGTCCGACCTG
M-CDKNA R	CCATGAGCGCATCGCAATC
M-P53 F	CTCTCCCCCGCAAAAGAAAAA
M-P53 R	CGGAACATCTCGAAGCGTTTA
M-PARP1 F	GGCAGCCTGATGTTGAGGT
M-PARP1 R	GCGTACTCCGCTAAAAAGTCAC
M-GAPDH F	AGGTCGGTGTGAACGGATTTG
M-GAPDH R	TGTAGACCATGTAGTTGAGGTCA
M-Cav1.2 F	CTACAGAAACCCATGTGAGCAT
M-Cav1.2 R	CAGCCACGTTGTCAGTGTTG
M-SERCA2a F	TGGAACAACCCGGTAAAGAGT
M-SERCA2a R	CACCAGGGGCATAATGAGCAG
M-NCX F	CTTCCCTGTTTGTGCTCCTGT
M-NCX R	AGAAGCCCTTTATGTGGCAGTA
M-PLB F	AAAGTGCAATACCTCACTCGC
M-PLB R	GGCATTTCAATAGTGGAGGCTC
M-RYR2 F	ACGGCGACCATCCACAAAG
M-RYR2 R	AAAGTCTGTTGCCAAATCCTTCT
M-IL-6 F	TAGTCCTTCCTACCCCAATTTCC
M-IL-6 R	TTGGTCCTTAGCCACTCCTTC
M-IL-1β F	ACCTGTGTCTTTCCCGTGGAC
M-IL-1β R	GGGAACGTCACACACCAGCA
M-Ki67 F	ATCATTGACCGCTCCTTTAGGT
M-Ki67 R	GCTCGCCTTGATGGTTCCT
M-NPPA F	GCTTCCAGGCCATATTGGAG
M-NPPA R	GGGGGCATGACCTCATCTT
M-NPPB F	GAGGTCACTCCTATCCTCTGG
M-NPPB R	GCCATTTCCTCCGACTTTTCTC
M-CDKN2A F	CGCAGGTTCTTGGTCACTGT
M-CDKN2A R	TGTTCACGAAAGCCAGAGCG
M-Gja1 F	ACAGCGGTTGAGTCAGCTTG
M-Gja1 R	GAGAGATGGGGAAGGACTTGT
M-Myh6 F	GCCCAGTACCTCCGAAAGTC
M-Myh6 R	GCCTTAACATACTCCTCCTTGTC
M-ACTC F	GGCTGTATTCCCCTCCATCG
M-ACTC R	CCAGTTGGTAACAATGCCATGT
si-M-CDKN1A	CCAGCCTGACAGATTTCTA

© 2021. Liu Y et al. Published online at http://www.aginganddisease.org/EN/10.14336/AD.2021.0502
