# Supplementary figures and images for: Atrial Fibrillation Underlies Cardiomyocyte Senescence and Contributes to Deleterious Atrial Remodeling during Disease Progression
Source: Aging Dis. 2022 Feb 1;13(1):298–312. doi: 10.14336/AD.2021.0619 (PMC8782549; doi:10.14336/AD.2021.0619)

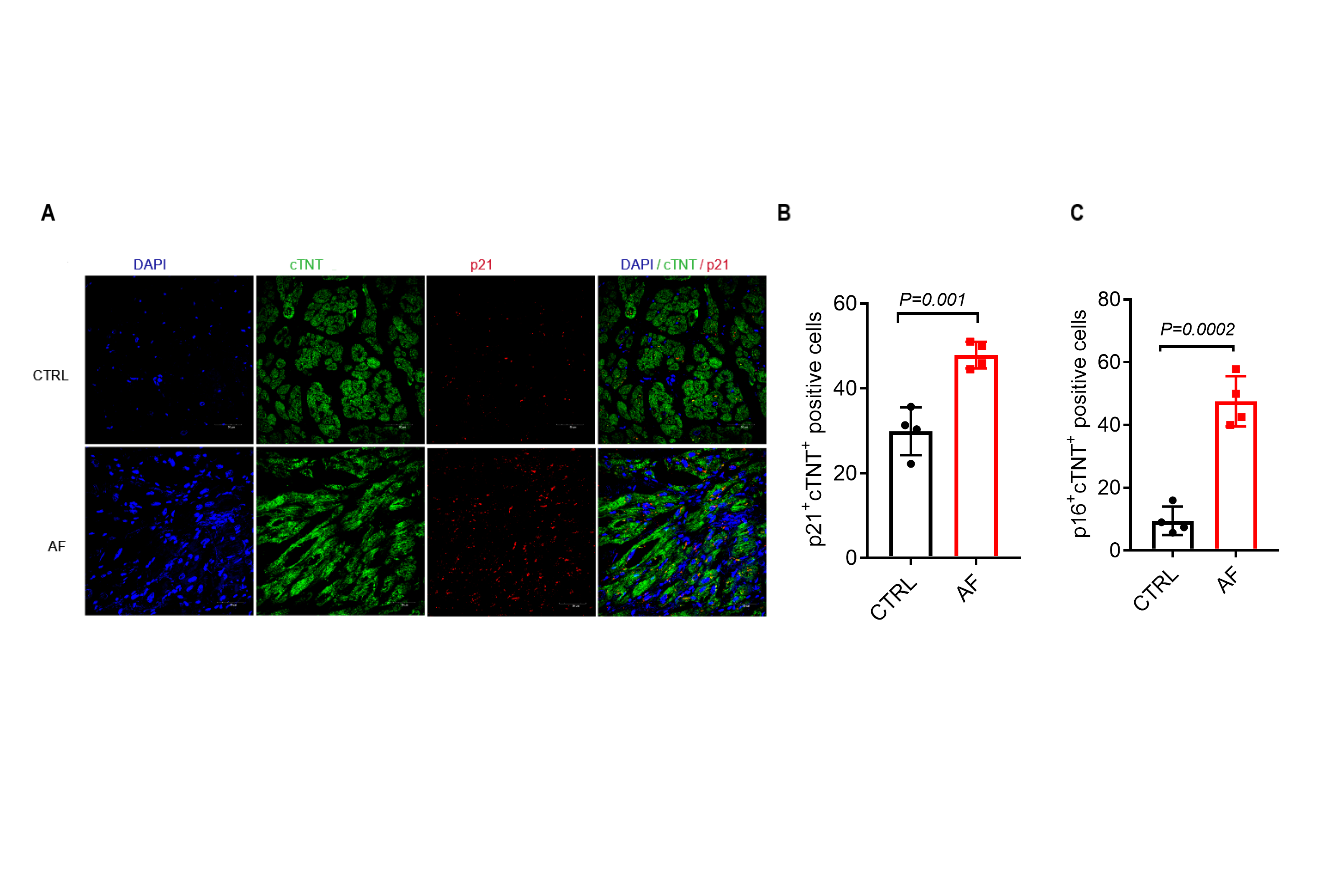

Supplement: Supplementary file 2 [file ad-13-1-298-s-g1.tif]

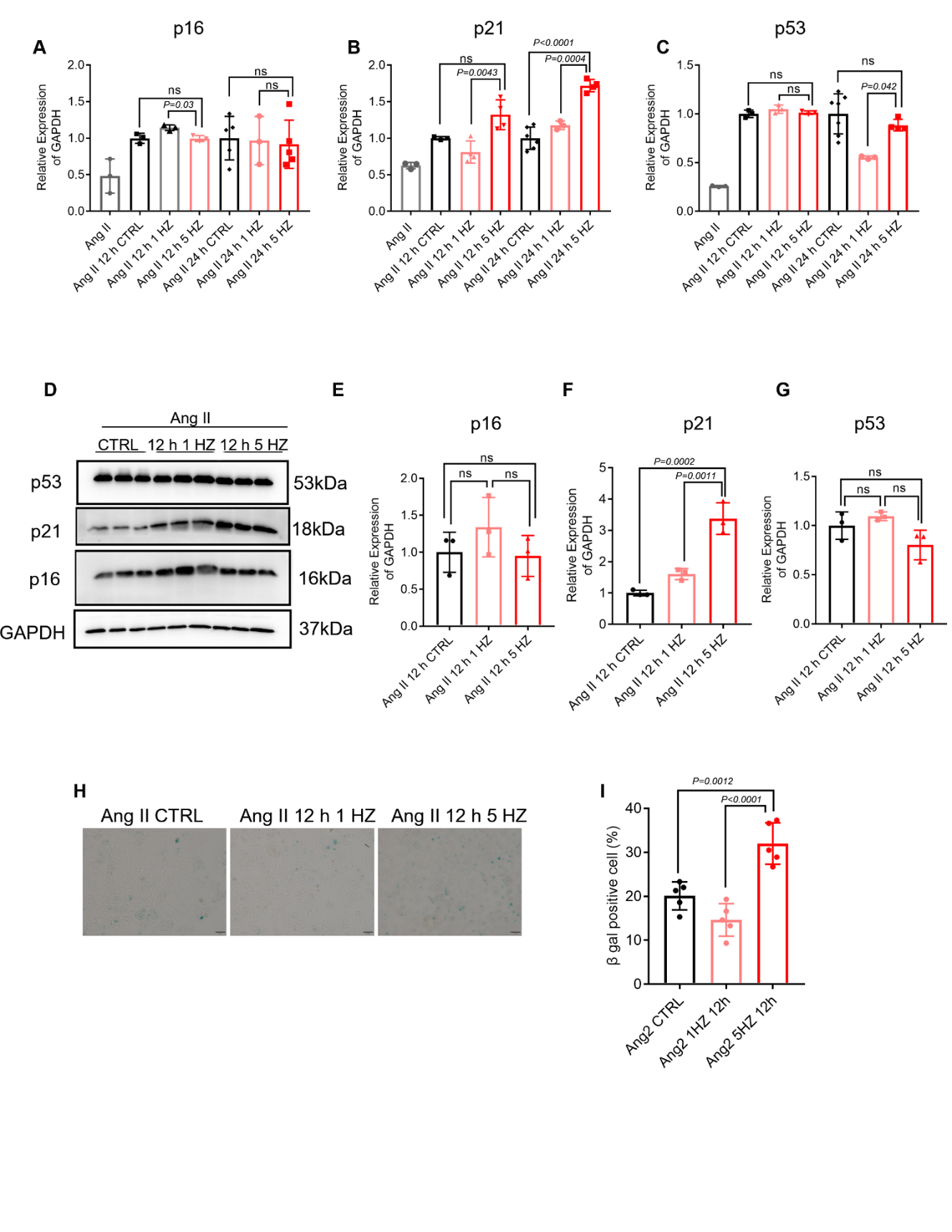

Supplement: Supplementary file 3 [file ad-13-1-298-s-g2.tif]

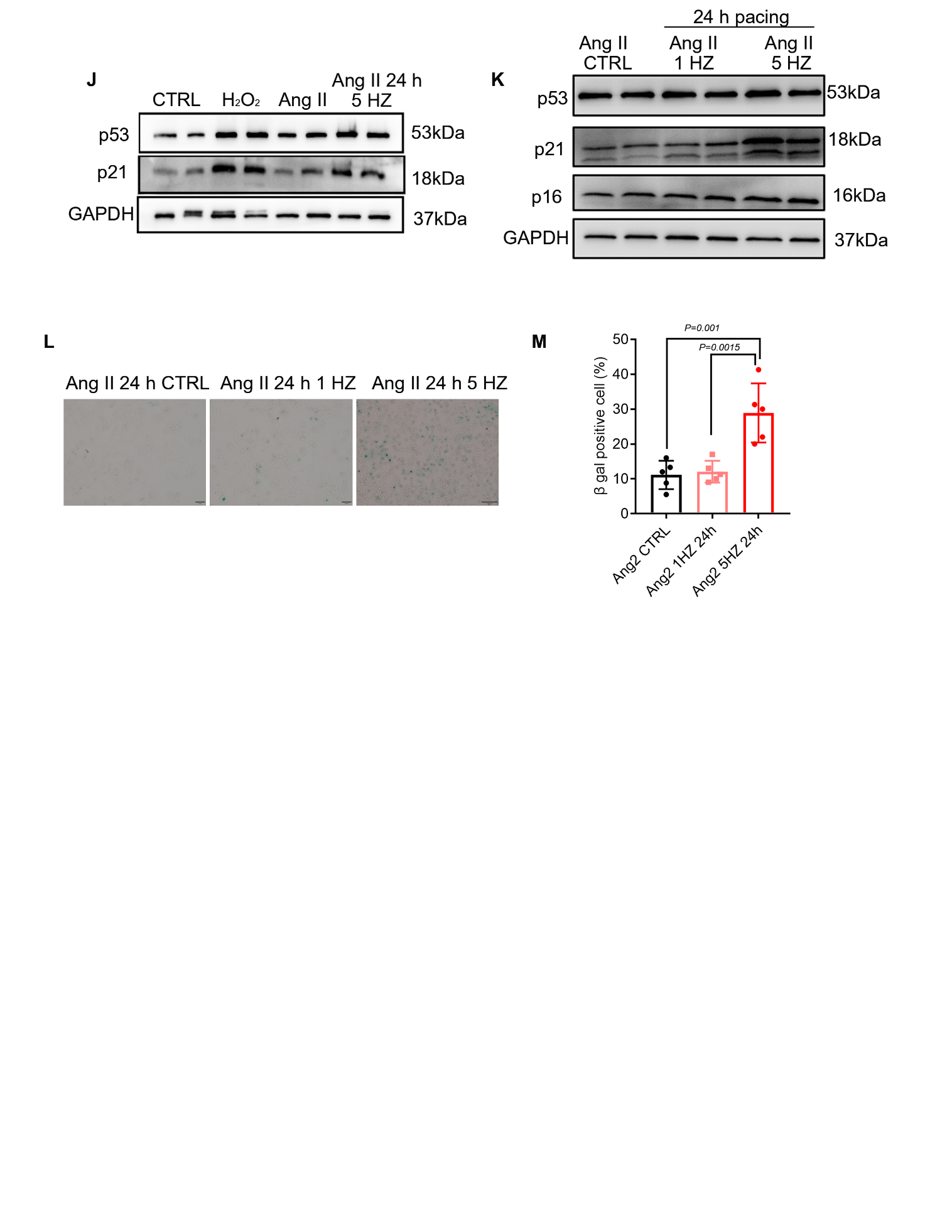

Supplement: Supplementary file 4 [file ad-13-1-298-s-g3.tif]

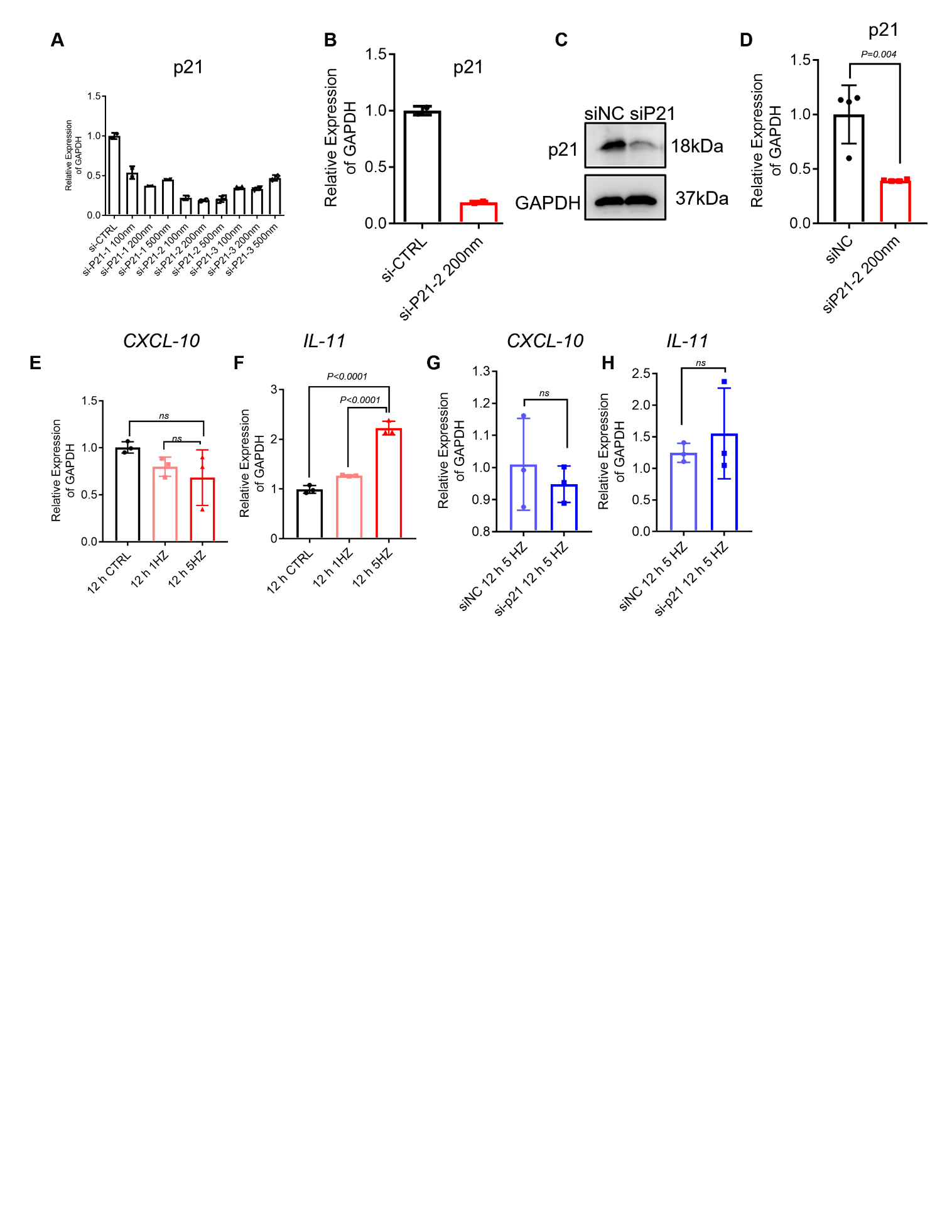

Supplement: Supplementary file 5 [file ad-13-1-298-s-g4.tif]

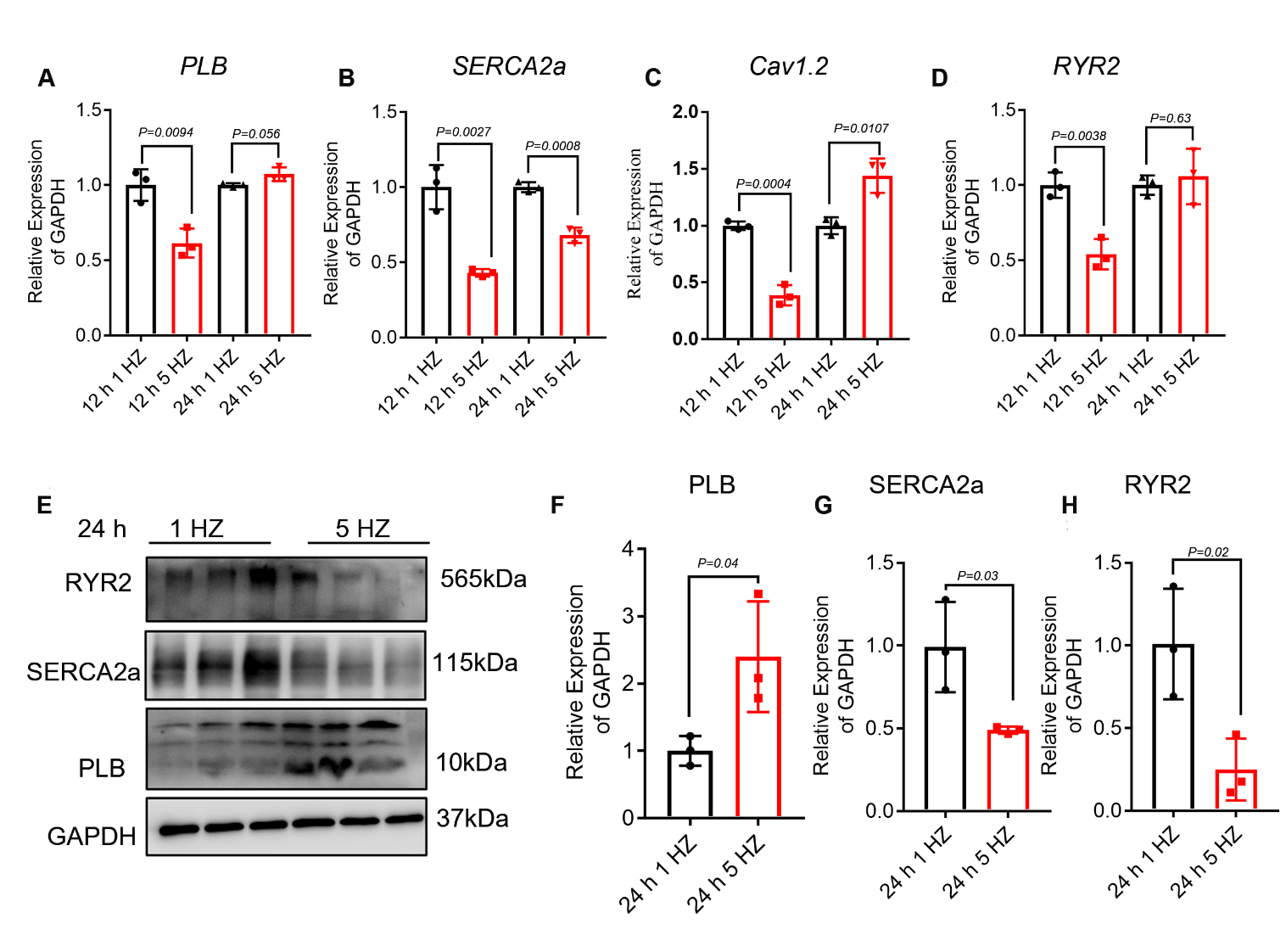

Supplement: Supplementary file 6 [file ad-13-1-298-s-g5.tif]

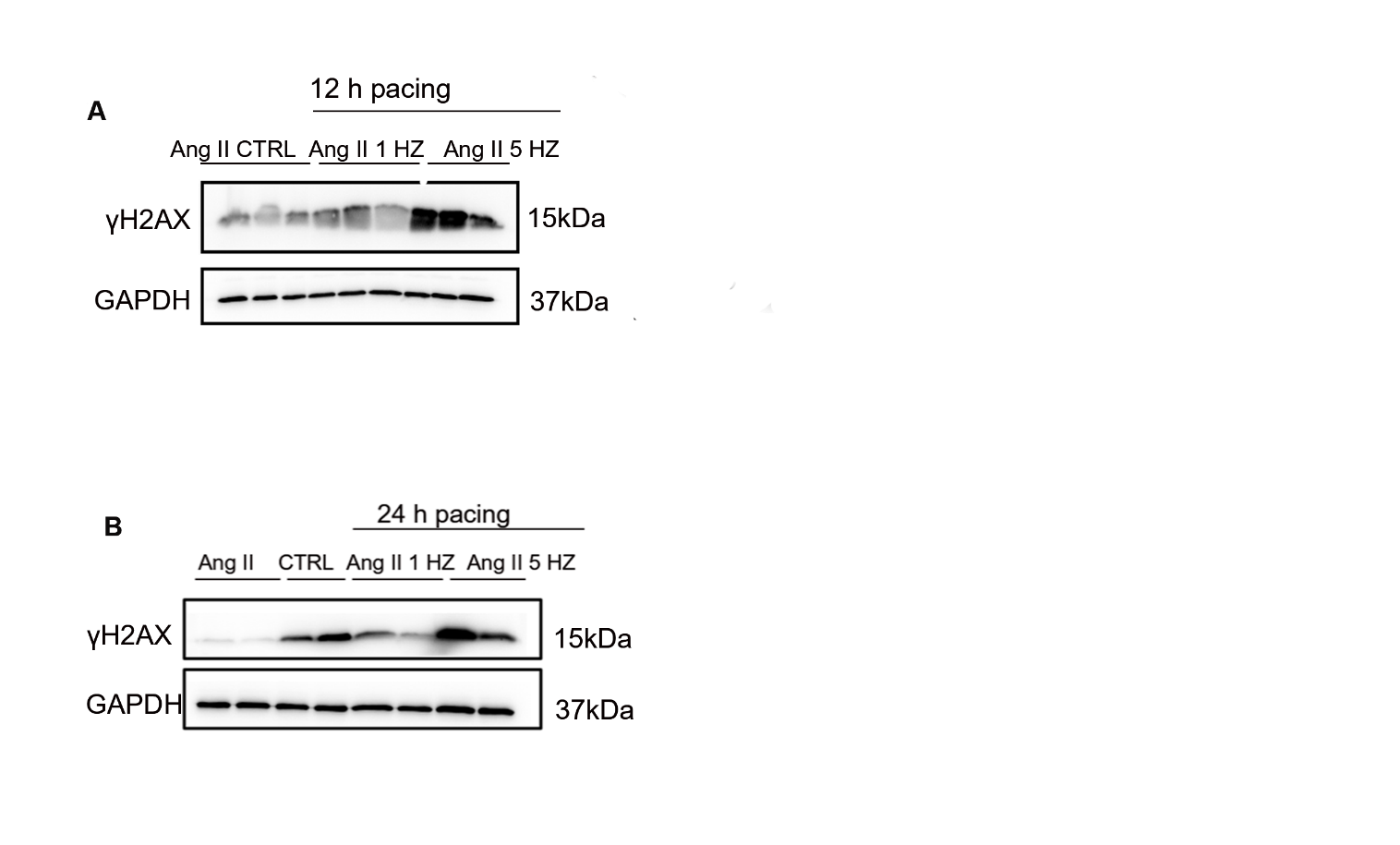

Supplement: Supplementary file 7 [file ad-13-1-298-s-g6.tif]
